# Risk of pneumonia in obstructive lung disease: A real-life study comparing extra-fine and fine-particle inhaled corticosteroids

**DOI:** 10.1371/journal.pone.0178112

**Published:** 2017-06-15

**Authors:** Samatha Sonnappa, Richard Martin, Elliot Israel, Dirkje Postma, Wim van Aalderen, Annie Burden, Omar S. Usmani, David B. Price, on behalf of the Respiratory Effectiveness Group, Small Airways Study Group

**Affiliations:** 1 Observational and Pragmatic Research Institute Pte Ltd, Singapore, Singapore; 2 Department of Respiratory Paediatrics, Rainbow Children’s Hospital, Bengaluru, India; 3 National Jewish Health and the University of Colorado Denver, Denver, Colorado, United States of America; 4 Pulmonary and Critical Care Division, Brigham & Women's Hospital, Harvard Medical School, Boston, MA, United States of America; 5 Department of Pulmonary Medicine and Tuberculosis, University of Groningen, University Medical Center Groningen, the Netherlands; 6 Department of Pediatric Respiratory Diseases, Emma Children’s Hospital AMC, Amsterdam, the Netherlands; 7 National Heart and Lung Institute, Imperial College London & Royal Brompton Hospital, London, United Kingdom; 8 Academic Primary Care, Division of Applied Health Sciences, University of Aberdeen, Aberdeen, United Kingdom; National and Kapodistrian University of Athens, GREECE

## Abstract

**Background:**

Regular use of inhaled corticosteroids (ICS) in patients with obstructive lung diseases has been associated with a higher risk of pneumonia, particularly in COPD. The risk of pneumonia has not been previously evaluated in relation to ICS particle size and dose used.

**Methods:**

Historical cohort, UK database study of 23,013 patients with obstructive lung disease aged 12–80 years prescribed extra-fine or fine-particle ICS. The endpoints assessed during the outcome year were diagnosis of pneumonia, acute exacerbations and acute respiratory events in relation to ICS dose. To determine the association between ICS particle size, dose and risk of pneumonia in unmatched and matched treatment groups, logistic and conditional logistic regression models were used.

**Results:**

14788 patients were stepped-up to fine-particle ICS and 8225 to extra-fine ICS. On unmatched analysis, patients stepping-up to extra-fine ICS were significantly less likely to be coded for pneumonia (adjusted odds ratio [aOR] 0.60; 95% CI 0.37, 0.97]); experience acute exacerbations (adjusted risk ratio [aRR] 0.91; 95%CI 0.85, 0.97); and acute respiratory events (aRR 0.90; 95%CI 0.86, 0.94) compared with patients stepping-up to fine-particle ICS. Patients prescribed daily ICS doses in excess of 700 mcg (fluticasone propionate equivalent) had a significantly higher risk of pneumonia (OR [95%CI] 2.38 [1.17, 4.83]) compared with patients prescribed lower doses, irrespective of particle size.

**Conclusions:**

These findings suggest that patients with obstructive lung disease on extra-fine particle ICS have a lower risk of pneumonia than those on fine-particle ICS, with those receiving higher ICS doses being at a greater risk.

## Introduction

Inhaled corticosteroids (ICS) are widely used in high doses in the management of obstructive lung diseases such as asthma[1] and chronic obstructive pulmonary disease (COPD).[2] Current asthma treatment guidelines recommend stepping up the ICS dose if lack of control persists.[1] Alternatively, the combination of low-dose ICS with long-acting β2-agonist (LABA) has been shown to achieve better asthma control, sparing patients higher doses of ICS.[3,4] In patients with COPD, low-dose ICS/LABA combination has been shown to reduce exacerbations, improve quality of life and lung function,[2] through an underlying complementary anti-inflammatory cellular action.[5,6] However there continues to be significant concern regarding inappropriate prescribing of high-dose ICS in patients with obstructive lung diseases, with untoward consequences for patients.[7–9]

Indeed, regular use of ICS has been linked to several systemic effects,[10] including a higher risk of pneumonia,[9,11–16] where it is thought that ICS exert an anti-inflammatory and immunosuppressive effect that could affect the pathogenesis of pneumonia.[17] Most randomized controlled trials (RCTs),[14,15,18] observational studies[11,19,20] and meta-analysis,[21,22] in patients with COPD suggest an increased risk of pneumonia with a dose-response relationship between ICS and pneumonia, although there is some evidence suggesting to the contrary.[23,24] This association is not as clear-cut in asthmatics; ICS are associated with a decreased risk of pneumonia based on RCTs, but observational studies suggest a higher risk of pneumonia.[25] Importantly though, all the published literature has assessed the risk of pneumonia only for conventional fine-particle ICS and not for extra-fine particle ICS in patients with obstructive lung disease.

While most ICS are fine particles with a mass median aerodynamic diameter (MMAD) of 2–4 microns, some extra-fine particle ICS formulations have been produced with a particle MMAD of 1.1 microns which improves airway deposition.[26–29] Two such ICS currently available are extra-fine beclometasone dipropionate (efBDP) and extra-fine ciclesonide (efCIC).[30] RCTs comparing the short-term efficacy of efBDP and efCIC to that of fine-particle ICS have found that extra-fine formulations offer equivalent efficacy when administered at half the dose of fine-particle ICS in both asthma and COPD.[31–34] Indeed, a rigorous dose response study has confirmed that efBDP provides significantly greater effects on lung function than comparable doses of fine-particle BDP. Of note, the improvements in obstructive lung disease symptoms and quality of life tend to be better with efBDP than fine-particle BDP at twice the dose,[35–40] suggesting that there may be clinically meaningful differences between the extra-fine particle and fine-particle formulations.

We therefore aimed to compare the risk of pneumonia and other adverse respiratory consequences between fine-particle ICS and extra-fine particle ICS in relation to their dose in patients with obstructive lung disease.

## Methods

### Study design

A historical cohort study was conducted in patients with obstructive lung disease in the UK consisting of a baseline and outcome period. The baseline period (for patient characterisation) was the one-year prior to the index prescription date, at which point asthma or COPD patients had their ICS therapy stepped-up (≥50% dose increase) as either extra-fine particle ICS (efBDP or efCIC) or fine-particle ICS (fluticasone propionate (FP) or BDP). Budesonide was not used in this investigation given the lack of or conflicting evidence that budesonide is associated with increased risk of pneumonia.[20,24,41,42] The study protocol was designed prior to data extraction by an independent steering committee and registered with the European Network of Centres for Pharmacoepidemiology and Pharmacovigilance (ENCePP registration number ENCEPP/EUPAS8832). Raw data were obtained from the Clinical Practice Research Datalink (CPRD), formerly known as the General Practice Research Database (www.cprd.com) and the UK Optimum Patient Care Research Database (OPCRD; www.optimumpatientcare.org). The CPRD is a governmental, not-for-profit research service, jointly funded by the NHS National Institute for Health Research (NIHR) and the Medicines and Healthcare products Regulatory Agency, a part of the Department of Health. All access and use of anonymised data via the CPRD are carefully controlled under UK and European law and the rules and regulations operating in the NHS. OPCRD has been reviewed and ethically approved by the NHS Health Research Authority to hold and process anonymised data as part of their service delivery (Research Ethics Committee reference: 15/EM/0150). The authors had no access to patient identifying information as part of this study and no informed consent was necessary as the data were anonymised.

### Statement of ethics approval

The study protocol was registered with the European Network of Centres for Pharmacoepidemiology and Pharmacovigilance (ENCePP, registration number: ENCEPP/EUPAS8832). Formal ethics and research management approval of the study protocol was obtained from the Anonymous Data Ethics Protocols and Transparency (ADEPT) committee and the Independent Scientific Advisory committee (ADEPTZZ015), which verify the scientific and ethical soundness of all research using OPCRD and CPRD data, respectively. The OPCRD has been approved by the Trent Multi Centre Research Ethics Committee for use in clinical research.

### Inclusion criteria

1. Aged: 12–80 years at the Index Prescription Date
2. Evidence of obstructive lung disease treatment defined as at least 2 prescriptions (either as fine-particle ICS or extra-fine particle ICS) for respiratory therapy at baseline (for patients in the ICS step-up cohort, including at least 1 prescription for ICS)
3. Continuation of therapy, defined as ≥2 respiratory prescriptions during the outcome year (i.e. ≥1 in addition to that prescribed at the Index Date)
4. Two years of continuous practice data comprising 1 year baseline data and 1 year of outcome data (up-to-standard data for CPRD patients)

### Exclusion criteria

Patients with any other chronic respiratory disease, at any time were excluded from the study.

### Outcome measures

#### Primary outcome

Diagnosis of pneumonia: (i) unconfirmed i.e. all unique patients with codes for pneumonia and, (ii) confirmed by chest radiograph or resulting in hospitalisation within one month of pneumonia diagnosis.

#### Secondary outcomes:

1. Exacerbation of asthma or COPD: An asthma *exacerbation* was defined as a course of oral corticosteroids (OCS), hospital admission, or emergency department attendance for asthma during the outcome year. For patients with COPD, exacerbations were defined as an acute course of OCS, antibiotics for a lower respiratory tract infection, or a recorded hospitalisation for COPD.
2. An acute respiratory event was defined as an occurrence of any of asthma or COPD related hospital admission, or emergency department attendance or acute use of OCS or antibiotics prescribed with lower respiratory consultation (consisting of the following: i) Lower respiratory read codes (including Asthma, COPD and LRTI Read codes); ii) Asthma/COPD review codes excluding any monitoring letter codes; iii) Lung function and/or asthma monitoring; iv) Any additional respiratory examinations, referrals, chest x-rays or events).

### Statistical analyses

Baseline characteristics of unmatched and matched patients prescribed either fine- or extra-fine particle ICS are described using summary statistics and compared using χ^2^ or Mann-Whitney *U* tests as appropriate.

### Outcome analysis

The adjusted odds of patients being coded for pneumonia in the outcome period was compared between unmatched and matched treatment groups using logistic and conditional logistic regression models. The dichotomous outcome for pneumonia coding/coding with confirmation was used as the dependent variable with treatment and potential confounding factors as explanatory variables (diabetes, baseline treatment and COPD). Both unadjusted and adjusted odds ratios (OR) with their respective 95% confidence intervals (CI) are reported. Results were adjusted for average daily ICS dose in the outcome and presented by categorised average daily ICS dose in the outcome period, to investigate the effect of ICS dose on developing pneumonia.

For the same population, two definitions of exacerbation of obstructive lung disease (asthma exacerbation and acute respiratory event) were used to compare respiratory outcomes between treatment groups, as indicators of clinical effectiveness. Event rates were compared between treatment groups using Poisson regression models. Exacerbation rates were the dependent variable, with treatment and potential confounding factors as explanatory variables (year of ICS step-up, acute courses of oral corticosteroids (0/1/2+) and categorised average daily ICS dose). The adjusted rate ratios (RR) with 95% confidence intervals are reported.

All analyses were carried out using IBM SPSS Statistics (version 21; SPSS Inc, Chicago, Ill), SAS software (version 9.3; SAS Institute Inc., Cary, NC, USA) and Microsoft Office Excel 2013 (Microsoft Corporation, Redmond, WA, USA). Statistically significant results are defined as p<0.05 and trends as 0.05 ≤ p<0.10.

Results are reported in accordance with STROBE guidelines for reporting cohort studies.

## Results

The results for unmatched groups are presented in the main paper and for matched groups in S1 File, S1 Fig and S1–S6 Tables.

Significant differences were observed between patients who received extra-fine versus fine-particle ICS in the demographics and baseline characteristics, as shown in Table 1. The ICS treatments prescribed to patients before and at step-up are shown in S1 Table in the supporting information. At step-up, 11952 (52%) patients were on FP; 2836 (12.3%) on BDP; 8117 (35.3%) on ef-BDP and 108 (0.4%) on ef-CIC.

**Table 1:** Demographic and clinical baseline characteristics for patients with obstructive lung disease prescribed fine- versus extra-fine particle ICS.

Demographic and clinical baseline characteristics		ICS particle-size		P-value^a^
		Patients (n = 23,013)		
		Fine-particle ICS (n = 14,788)	Extra-fine particle ICS (n = 8,225)	
**Demographics**				
Sex, female		9046 (61)	4871 (59)	0.004
Age at ICS step-up date (index date), mean (SD)		44 (17)	44 (18)	0.018
Baseline weight BMI (kg/m^2^), mean (SD)		28 (7)	29 (7)	0.001
Year of step-up date (index date), median (IQR)		2002 (1998, 2007)	2006 (2003, 2008)	<0.001
Smoking^b^	Non-smokers	8679 (59)	4873 (59)	0.024
	Current smokers	3474 (24)	1904 (23)	
	Ex-smokers	2476 (17)	1392 (17)	
Respiratory diagnosis	None	174 (1.2)	114 (1.4)	NA
	Asthma/ no COPD	11516 (77.9)	7171 (87.2)	
	COPD/ no asthma	197 (1.3)	93 (1.1)	
	Asthma and COPD	2901 (19.6)	847 (10.3)	
**Comorbidities and Therapy**				
Rhinitis diagnosis and/or therapy^c^		6175 (42)	2754 (34)	<0.001
GERD diagnosis and/or drugs^d^		3827 (26)	2115 (26)	0.784
Ischaemic heart disease diagnosis^e^		1084 (7)	433 (5)	<0.001
Coding for pneumonia^f^		73 (0.5)	22 (0.3)	0.010
Confirmed coding for pneumonia^g^		25 (0.2)	12 (0.1)	0.674
**Baseline characteristics**				
Acute oral corticosteroid courses^h^	0	8542 (58)	6008 (73)	<0.001
	1	3103 (21)	1420 (17)	
	2+	3143 (21)	797 (10)	
Antibiotics prescribed with lower respiratory consultation^i^	0	9027 (61)	5440 (66)	<0.001
	1	3060 (21)	1667 (20)	
	2+	2701 (18)	118 (14)	
ICS dose at date of step-up (index date), median (IQR)^j^		1000 (500, 1000)	400 (200, 400)	<0.001
Average ICS daily dose (μg), median (IQR)^j^		214 (82, 438)	110 (55, 219)	<0.001

Data are n (%) unless otherwise stated. BMI: body mass index; COPD: chronic obstructive pulmonary disease; GERD: gastroesophageal reflux disease; ICS: inhaled corticosteroids; IQR: interquartile range; NA: not applicable.

^a^Conditional logistic regression.

^b^Matching variable.

^c^Read code at any time and/or prescription for nasal spray during baseline or outcome analysis period.

^d^Read code and/or drugs for GERD (BNF 1.3.5) at any time.

^e^Read code at any time.

^f^Pneumonia coding defined as a Read code for pneumonia, at any time.

^g^Pneumonia coding confirmed by x-ray or hospitalization.

^h^Acute oral corticosteroid courses were defined as all courses that are definitely not maintenance therapy, and/or all courses where dosing instructions suggest exacerbation treatment (e.g. 6,5,4,3,2,1 reducing, or 30μg as directed), and/or all courses with no dosing instructions, but unlikely to be maintenance therapy with a code for asthma or a lower respiratory event, where “maintenance therapy” is defined as: daily dosing instructions of <10μg prednisolone or prescriptions for 1mg prednisolone tablets.

^i^Lower respiratory consultations consist of the following: a) lower respiratory Read codes (including asthma, COPD and LRTI Read codes); b) asthma/COPD review codes excluding any monitoring letter codes; c) lung function and/or asthma monitoring; d) any additional respiratory examinations, referrals, chest x-rays or events.

^j^*Fluticasone* propionate (FP) equivalent.

**Fig 1:**
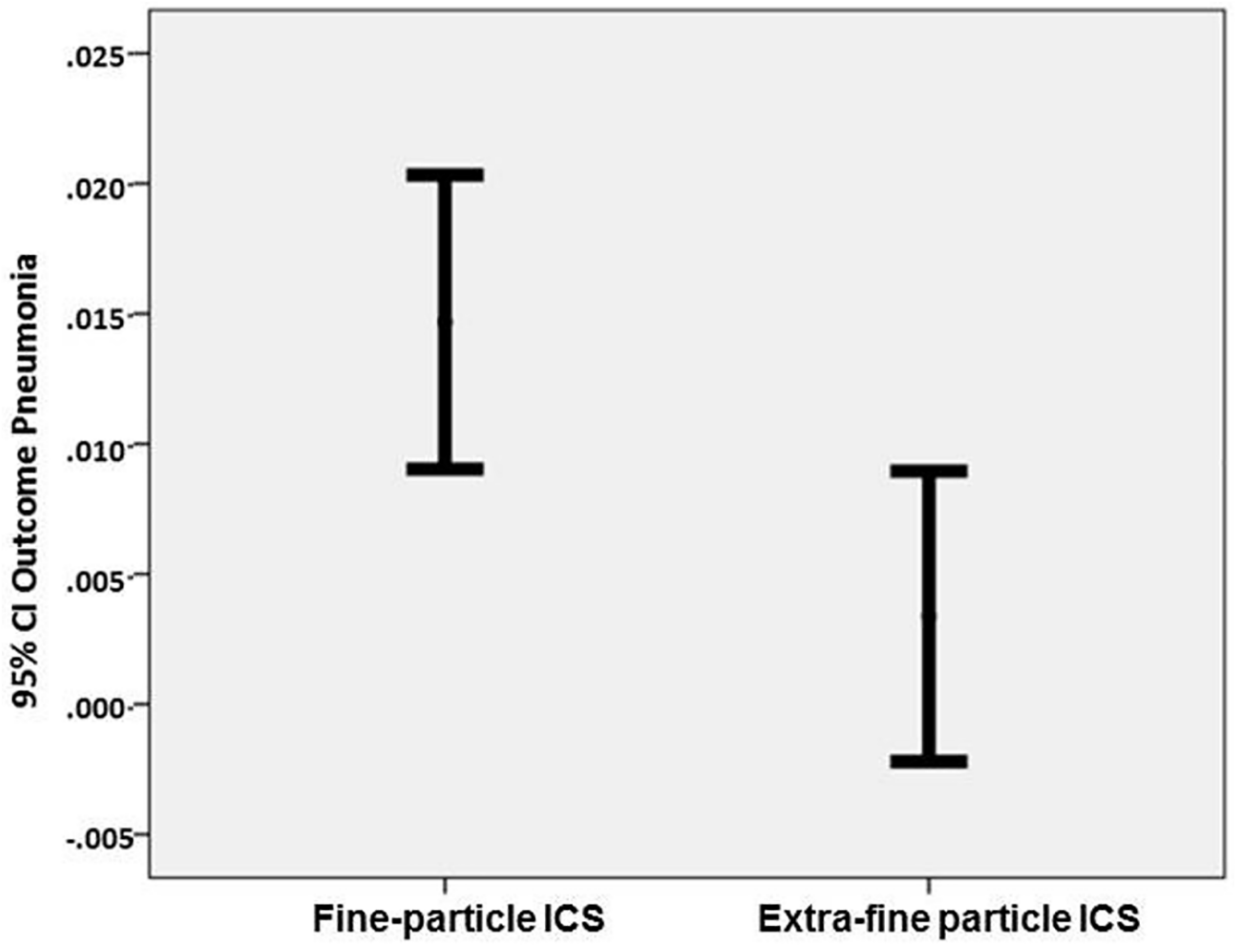
Probability (95% CI) of pneumonia in outcome period by treatment group.

Patients stepping-up their ICS therapy to extra-fine particle ICS were significantly less likely to be coded for pneumonia (OR [95% CI] 0.60 [0.37, 0.97]; p<0.011) compared to those stepping-up to fine-particle ICS, having adjusted for confounders (Table 2; Fig 1).

**Table 2:** Pneumonia diagnosis by treatment group.

Pneumonia diagnosis	By treatment group		Total	P-value^a^
	Fine-particle	Extra-fine particle		
Yes, n (%)	73 (0.5)	22 (0.3)	95 (0.4)	0.011
No, n (%)	14715 (99.5)	8203 (99.7)	22918 (99.6)	
Total, n (%)	14788 (100)	8225 (100)	23013 (100)	
Odds ratio adjusted for baseline confounders^b^	1.00	0.60 (0.37, 0.97)		

^a^Fisher’s exact 2-sided test.

^b^Adjusted for baseline diagnosis for pneumonia (Y/N), diabetes diagnosis or therapy at baseline (Y/N) and COPD diagnosis (ever) (Y/N).

Furthermore, patients stepping-up to extra-fine particle ICS were significantly less likely to experience acute exacerbations (RR [95%CI] 0.91 [0.85, 0.97]); p<0.001) (Table 3) and acute respiratory events (RR [95%CI] 0.90 [0.86, 0.94]; p<0.001) (Table 4) compared with patients stepping-up to fine-particle ICS, having adjusted for confounders.

**Table 3:** ATS/ERS exacerbations in outcome period by treatment group.

ATS/ERS exacerbations in outcome period	By treatment group		Total	P-value^a^
	Fine-particle	Extra-fine particle		
**0, n (%)**	10087 (68.2)	6547 (79.6)	16634 (72.3)	<0.001
**1, n (%)**	2579 (17.4)	1099 (13.4)	3678 (16.0)	
**2+, n (%)**	2122 (14.3)	579 (7.0)	2701 (11.7)	
**Total, n (%)**	14788 (100)	8225 (100)	23013 (100)	
**Rate ratio adjusted for baseline confounders**^**b**^	1.00	0.91 (0.85, 0.97)		

ATS: American Thoracic Society; ERS: European Respiratory Society.

^a^χ^2^ test.

^b^Adjusted for year of ICS step-up, acute courses of oral corticosteroids (0/1/2+) and average daily ICS dose (categorised).

**Table 4:** Acute respiratory events in outcome period by treatment group.

Acute respiratory events in outcome	By treatment group		Total	P-value^a^
	Fine-particle	Extra-fine particle		
**0, n (%)**	7,936 (53.7)	5,353 (65.1)	13,289 (57.7)	<0.001
**1, n (%)**	3,357 (22.7)	1,715 (20.9)	5,072 (22)	
**2+, n (%)**	3,495 (23.6)	1,157 (14.1)	4,652 (20.2)	
**Total, n (%)**	14,788 (100)	8,225 (100)	23,013 (100)	
**Rate ratio adjusted for baseline confounders**^**b**^	1.00	0.90 (0.86, 0.94)		

^a^χ^2^ test.

^b^Adjusted for year of ICS step-up, Charlson Comorbidity Index Score (categorised), acute courses of oral corticosteroids (0/1/2) and average daily ICS dose (categorised).

**Fig 2:**
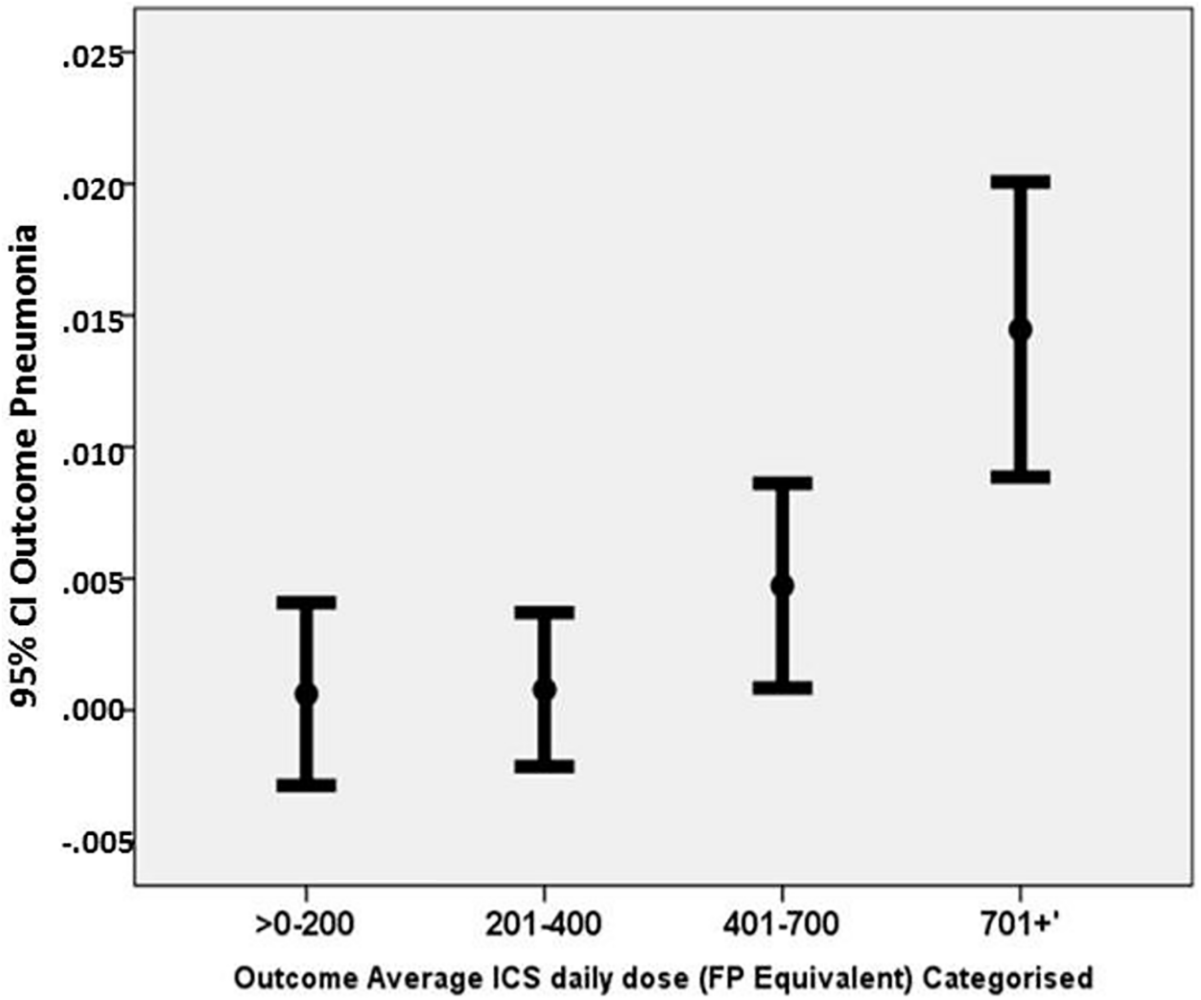
Probability (95% CI) of pneumonia in outcome period by average daily consumed ICS dose during outcome period.

An analysis by average daily ICS dose suggested patients receiving daily ICS doses in excess of 700 mcg (FP equivalent) were significantly more likely (OR [95%CI] 2.38 [1.17, 4.83]); p<0.001) (Table 5; Fig 2) to be coded for pneumonia compared with patients prescribed lower doses, irrespective of particle size (S1 Fig in the supporting information).

**Table 5:** Pneumonia diagnosis by average daily consumed ICS dose during outcome period.

Pneumonia diagnosis	By average daily dose (mcg FP equivalent dose)				Total	P-value^a^
	>0–200	201–400	401–700	701+		
Yes, n (%)	10 (0.2)	15 (0.2)	24 (0.4)	46 (0.8)	95 (0.4)	<0.001
No, n (%)	4444 (99.8)	6471 (99.8)	6141 (99.6)	5862 (99.2)	22918 (99.6)	
Total, n (%)	4454 (100)	6486 (100)	6165 (100)	5908 (100)	23013 (100)	
Odds ratio adjusted for baseline confounders^b^	1.00	0.92 (0.41, 2.05)	1.36 (0.64, 2.87)	2.38 (1.17, 4.83)		

ICS: inhaled corticosteroids; FP: fluticasone propionate.

^a^χ^2^ test.

^b^Adjusted for baseline diagnosis for pneumonia (Y/N), diabetes or therapy at baseline (Y/N) and COPD diagnosis (ever) (Y/N).

Results for analysis of 6636 uniquely matched patient pairs stepping-up to extra-fine particle ICS compared to those stepping-up to fine-particle ICS are presented in the supporting information. S2 Table in the supporting information shows the patient flow for matching and S3 Table the demographics and baseline characteristics. Matched patients stepping-up to extra-fine particle ICS were significantly less likely (OR [95% CI] 0.50 [0.27, 0.93]; p = 0.028) to be coded for pneumonia compared with patients stepping-up to fine-particle ICS, before adjusting for confounders. After adjusting for COPD diagnosis, significance was not maintained, although a trend for a lower incidence of pneumonia (OR [95% CI] 0.50 [0.25, 1.01]; p = 0.054) (S4 Table in the supporting information) was seen in matched patients stepping-up to extra-fine particle ICS.

Matched patients stepping-up to extra-fine particle ICS were also significantly less likely to experience acute exacerbations (RR [95%CI] 0.90 [0.84, 0.97]); p<0.001) (S5 Table in the supporting information) and acute respiratory events (RR [95%CI] for 0.90 [0.86, 0.95]; p<0.001) (S6 Table in the supporting information) compared with patients stepping-up to fine-particle ICS, having adjusted for confounders.

## Discussion

Our data show that patients with obstructive lung disease using fine-particle ICS (FP or BDP) stepping-up their ICS therapy to extra-fine particle ICS (efBDP or efCIC) are significantly less likely to have pneumonia in unmatched patients with a trend for lower incidence in matched patients after adjusting for COPD diagnosis. Furthermore, patients on extra-fine ICS are significantly less likely to experience adverse respiratory outcomes compared to patients stepping-up to fine-particle ICS on both unmatched and matched analysis. Patients on daily ICS doses in excess of 700 mcg (fluticasone propionate equivalent) have a significantly increased risk of pneumonia compared with patients on lower doses, irrespective of particle size. To the best of our knowledge, this is the first study to compare the risk of pneumonia between extra-fine and fine-particle ICS in patients with obstructive lung disease in relation to ICS dose.

Our findings are particularly pertinent in patients with chronic airway colonisation by pathogenic bacteria, which has been identified as a risk factor for COPD disease exacerbations.[43] Additionally, and of interest since the largest proportion of patients had solely an asthma diagnosis while results persisted after adjusting for COPD, our findings suggest that higher ICS doses may be driving the higher incidence rates of pneumonia seen in patients with obstructive airway disease, as previously observed in both asthma[9] and COPD patients.[19]

The pathophysiological mechanisms that contribute to an increased susceptibility to pneumonia in patients treated with ICS are unclear. In murine models, ICS have been shown to significantly increase alveolar macrophage efferocytosis (uptake of apoptotic cells by alveolar macrophages), thereby reducing their ability to combat microbes, including *Streptococcus pneumoniae*, the most common cause of community acquired pneumonia in patients with COPD.[44] A recent study in a cohort of children with persistent asthma taking daily ICS showed nearly four times greater oropharyngeal colonization with *Streptococcus pneumoniae* compared to children not receiving ICS,[45] which may increase the risk of having pneumococcal respiratory infections. Several studies have demonstrated an intra-class difference between both mono-component ICS and fixed combinations of ICS/LABA with regard to the risk of pneumonia and pneumonia related events in COPD patients.[20,22,24,41] The risk of patients with COPD developing serious pneumonia is particularly elevated and dose related with fluticasone use and much lower with budesonide.[11] Although there have been no studies directly comparing the effects of fluticasone and budesonide on host defence, differences are likely related to their contrasting pharmacokinetic and pharmacodynamic properties. To prevent any confounding by differences between FP and budesonide, we therefore excluded patients using budesonide in our current analyses.

ICS are reported to be overused in the treatment of COPD[7,46–48] contrary to current guidelines that recommend reserving the use of ICS, in addition to a maintenance therapy with long-acting β_2_-agonists (LABA) and/or a LABA and long-acting muscarinic antagonists, for patients with severe or very severe airflow limitation and/or two or more exacerbations per year.[2] Some studies have shown that ICS discontinuation does not lead to significant adverse effects, such as an excess of COPD exacerbations in subjects without an asthma component to their COPD. [49–51] Furthermore, a recent Cochrane review supports current guidelines advocating LABA as frontline therapy for COPD, with regular ICS as an adjunct only in patients experiencing frequent exacerbations.[52]

Similarly in patients with asthma, when low-moderate doses of ICS fail to achieve symptom control, ICS/LABA combination therapy has been shown to improve symptom control, reduce acute exacerbations and improve lung function.[3,4,53,54] In mild-to-moderate asthma, ICS dose-dependent improvements in markers of control occur, but the dose-response profile is shallow and use of higher ICS doses does not increase the efficacy of these drugs and comes at an expense of an increase in the incidence of side effects.[54–56] Extra-fine particle ICS have a favourable safety profile with decreased local and systemic exposure, and are associated with improved clinical outcomes when compared to equivalent ICS doses of larger sized aerosols in patients with asthma and COPD,[31–34,39,40]. In these patients extra-fine ICS formulations can perhaps be used at lower doses without compromising on symptom control.

Our study has strengths and limitations. This study drew on two large primary care databases, the CPRD and OPCRD, allowing us to obtain a large sample of 23,013 patients with obstructive lung disease. There was no difference in the incidence of pneumonia confirmed radiologically or by hospitalisation between COPD and asthma patients, and there is a potential that diagnostic misclassification could have arisen from acute COPD exacerbation episodes, since the patient symptoms may have been similar to those of patients with pneumonia. Nevertheless, as patients receiving extra-fine particle ICS had significantly fewer adverse respiratory outcomes compared with fine-particle ICS, we believe that diagnostic misclassification would not have had a substantial effect on our findings. We clubbed patients with asthma and COPD under one umbrella term of obstructive lung disease, having recognised *a priori* that the data would not be adequately powered for a subgroup analysis of the individual cohorts. However, the different pathophysiology, inflammatory profile and response to treatment in patients with asthma and COPD could partly explain the statistical insignificance of the primary endpoint after adjusting for COPD diagnosis as our cohort was predominantly made up of asthmatics. “Furthermore, although we excluded patients using budesonide from the analysis, an ideal comparative study would be between fine-particle and extra-fine particle fluticasone propionate if and when such a formulation became available”.

Moreover, the seasonal and yearly variations seen in the distribution of community acquired pneumonia and our observation period of one-year may have been inadequate to determine a true risk. The extent of the advantage of extra-fine particle ICS over fine-particle ICS may have been overestimated in the unmatched analysis as the matched pairs only showed a trend for a lower risk of pneumonia with extra-fine particle ICS after adjusting for the diagnosis of COPD. The main benefit of a matched cohort analysis is a probable increase in efficiency and the selection of the matching variables is vital.[57] In practice, it is difficult to establish the strength of the association between matching variables, the exposure of interest and the outcome. In real-life situations an unmatched design is probably more representative of the patient population and therefore less biased and more generalizable. We have therefore reported both unmatched and matched analysis results.

In summary, we have shown important findings relevant to prescribing clinicians in the day-to-day management of patients with obstructive lung disease, where an increased risk of pneumonia and higher rates of adverse respiratory events are more likely with fine-particle ICS compared to extra-fine particle ICS. The benefit-risk ratio is an important measure of safety of drugs and the additional respiratory morbidity observed in our study with higher doses of ICS prescribed more frequently to those on fine-particle ICS favours extra-fine particle ICS for the treatment of obstructive lung diseases, particularly at higher doses. Indeed, our data support guideline directed management[1] that recommend patients should be maintained at the lowest possible dose of ICS, which can potentially be achieved more easily with extra-fine particle ICS formulations.

## Supporting information

S1 Fig Probability (95% CI) of pneumonia diagnosis by average daily consumed ICS dose during outcome period by particle size. (JPG)

S1 Table ICS prescribed to patients before and at step-up. (DOCX)

S2 Table Patient flow diagram for matching patients with obstructive lung disease on fine-particle vs. extra-fine particle. (DOCX)

S3 Table Demographic and clinical baseline characteristics for matched patients with obstructive lung disease prescribed fine- versus extra-fine particle ICS. (DOCX)

S4 Table Adjusted outcome results–pneumonia diagnosis by treatment group: fine vs. extra-fine particle in matched patients. (DOCX)

S5 Table Adjusted outcome results–ATS/ERS exacerbations by treatment group: fine vs. extra-fine particle in matched patients. (DOCX)

S6 Table Adjusted outcome results–acute respiratory events by treatment group: fine vs. extra-fine particle in matched patients. (DOCX)

S1 File Online supplement. (DOCX)
