# Supplementary figures and images for: Risk of pneumonia in obstructive lung disease: A real-life study comparing extra-fine and fine-particle inhaled corticosteroids
Source: PLoS One. 2017 Jun 15;12(6):e0178112. doi: 10.1371/journal.pone.0178112 (PMC5472262; doi:10.1371/journal.pone.0178112)

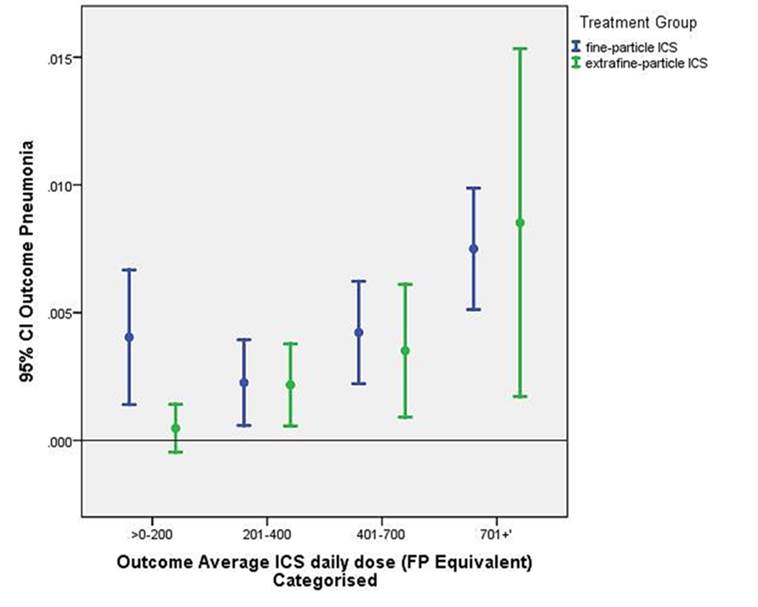

Supplement: S1 Fig — (JPG) [file pone.0178112.s001.jpg]
