# Supplementary material for: Risk of pneumonia in obstructive lung disease: A real-life study comparing extra-fine and fine-particle inhaled corticosteroids
Source: PLoS One. 2017 Jun 15;12(6):e0178112. doi: 10.1371/journal.pone.0178112 (PMC5472262; doi:10.1371/journal.pone.0178112)
Supplement: S1 Table — (DOCX) [file pone.0178112.s002.docx]

S1 Table. Inhaled corticosteroids prescribed to patients before and at step-up.

| **Last drug prescribed prior to step-up** | **Drug substance at step-up** | | | | **Total** |
| --- | --- | --- | --- | --- | --- |
|  | **Fluticasone** | **Clenil** | **QVAR** | **Ciclesonide** |  |
| Fluticasone | 5694 (47.6) | 25 (0.9) | 121 (1.5) | 11 (10.2) | 5851 (25.4) |
| Fine-particle BDP | 5309 (44.4) | 2576 (90.8) | 3832 (47.2) | 37 (34.3) | 11754 (51.1) |
| Mometasone | 10 (0.1) | 1 (0) | 9 (0.1) | 2 (1.9) | 22 (0.1) |
| Budesonide | 704 (5.9) | 99 (3.5) | 231 (2.8) | 4 (3.7) | 1038 (4.5) |
| QVAR | 224 (1.9) | 129 (4.5) | 3914 (48.2) | 7 (6.5) | 4274 (18.6) |
| Ciclesonide | 11 (0.1) | 6 (0.2) | 10 (0.1) | 47 (43.5) | 74 (0.3) |
| Total | 11952 (100) | 2836 (100) | 8117 (100) | 108 (100) | 23013 (100) |

BDP: beclometasone dipropionate.
