# Supplementary material for: Risk of pneumonia in obstructive lung disease: A real-life study comparing extra-fine and fine-particle inhaled corticosteroids
Source: PLoS One. 2017 Jun 15;12(6):e0178112. doi: 10.1371/journal.pone.0178112 (PMC5472262; doi:10.1371/journal.pone.0178112)
Supplement: S2 Table — (DOCX) [file pone.0178112.s003.docx]

**S2 Table****. Patient flow diagram for matching patients with obstructive lung disease on fine-particle vs. extra-fine particle.**

|  | **Fine-particle ICS** | | **Extra-fine particle ICS** | |
| --- | --- | --- | --- | --- |
|  | **n = 14,788** | | **n = 8,225** | |
|  | **# Remaining** | **# Lost** | **# Remaining** | **# Lost** |
| Sex | 14788 | 0 | 8225 | 0 |
| Age | 14788 | 0 | 8225 | 0 |
| Baseline pneumonia | 14781 | 7 | 8225 | 0 |
| Smoking status | 14761 | 20 | 8221 | 4 |
| OCS courses | 14697 | 64 | 8206 | 15 |
| Antibiotics | 14655 | 42 | 8190 | 16 |
| Average daily ICS dose | 13327 | 1328 | 8157 | 33 |
| Year of IPD | 10376 | 2951 | 7967 | 190 |

ICS: inhaled corticosteroids; IPD: index prescription date; OCS: oral corticosteroids.
