# Supplementary material for: Risk of pneumonia in obstructive lung disease: A real-life study comparing extra-fine and fine-particle inhaled corticosteroids
Source: PLoS One. 2017 Jun 15;12(6):e0178112. doi: 10.1371/journal.pone.0178112 (PMC5472262; doi:10.1371/journal.pone.0178112)
Supplement: S3 Table — (DOCX) [file pone.0178112.s004.docx]

S3 Table. Demographic and clinical baseline characteristics for matched patients with obstructive lung disease prescribed fine- vs. extra-fine particle ICS.

| **Demographic and clinical baseline characteristics** | | **ICS particle-size** | | **P-value**^a^ |
| --- | --- | --- | --- | --- |
|  |  | **Patients (n=13272)** | |  |
|  | | **Fine-particle ICS (n=6636)** | **Extra-fine particle ICS (n=6636)** |  |
| **Demographics** | | | | |
| Sex, female^b^ | | 3936 (59) | 3936 (59) | NA |
| Age at ICS step-up date (index date), mean (SD)^b^ | | 43 (18) | 43 (18) | 0.060 |
| Baseline weight BMI (kg/m^2^), mean (SD) | | 28 (7) | 28 (7) | 0.739 |
| Year of step-up date (index date), median (IQR) | | 2006 (2002, 2008) | 2006 (2003, 2008) | <0.001 |
|  |  |  |  |  |
| Smoking^b^ | Unknown | 29 (0.4) | 29 (0.4) |  |
|  | Non-smokers | 4074 (61.4) | 4074 (61.4) | 0.703 |
|  | Current smokers | 1504 (22.7) | 1504 (22.7) |  |
|  | Ex-smokers | 1029 (15.5) | 1029 (15.5) |  |
| **Comorbidities and Therapy** | | | | |
| Rhinitis diagnosis and/or therapy^c^ | | 2511 (37.8) | 2212 (33.3) | <0.001 |
| GERD diagnosis and/or drugs^d^ | | 1637 (24.7) | 1625 (24.5) | 0.799 |
| Ischaemic heart disease diagnosis^b,e^ | | 349 (5.3) | 340 (5.1) | 0.716 |
| Coding for pneumonia^f^ | | 3 (0) | 3 (0) | NA |
| Confirmed coding for pneumonia^g^ | | 0 (0) | 1 (0) | 0.610 |
| Charlson Comorbidity Index score | 0 | 2618 (39.5) | 2576 (38.8) | 0.174 |
|  | 1-4 | 3472 (52.3) | 3462 (52.2) |  |
|  | 5+ | 546 (8.2) | 598 (9) |  |
| **Baseline characteristics** | | | | |
| Acute oral corticosteroid courses^b,h^ | 0 | 4899 (73.8) | 4899 (73.8) | NA |
|  | 1 | 1099 (16.6) | 1099 (16.6) |  |
|  | 2+ | 638 (9.6) | 638 (9.6) |  |
| Antibiotics prescribed with lower respiratory consultation^b,i^ | 0 | 4505 (67.9) | 4505 (67.9) | NA |
|  | 1 | 1279 (19.3) | 1279 (19.3) |  |
|  | 2+ | 852 (12.8) | 852 (12.8) |  |
| ICS dose at date of step-up (index date), median (IQR)^j^ | | 500 (250, 1000) | 400 (200, 400) | <0.001 |
| Average ICS daily dose (µg), median (IQR)^j^ | | 115 (55, 219) | 110 (55, 219) | 0.007 |
|  |  |  |  |  |
| Blood eosinophil level (μ/L) | <400 | 1296 (71.2) | 1637 (75.6) | 0.031 |
|  | >400 | 523 (28.8) | 529 (24.4) |  |

Data are n (%) unless otherwise stated.

BMI: body mass index; COPD: chronic obstructive pulmonary disease; GERD: gastroesophageal reflux disease; ICS: inhaled corticosteroids; IQR: interquartile range; PEF: percentage of forced expiratory volume; NA: not applicable.

^a^Conditional logistic regression.

^b^Matching variable.

^c^Read code at any time and/or prescription for nasal spray during baseline or outcome analysis period.

^d^Read code and/or drugs for GERD (BNF 1.3.5) at any time.

^e^Read code at any time.

^f^Pneumonia coding defined as a Read code for pneumonia, at any time.

^g^Pneumonia coding confirmed by x-ray or hospitalization.

^h^Acute oral corticosteroid courses were defined as all courses that are definitely not maintenance therapy, and/or all courses where dosing instructions suggest exacerbation treatment (e.g. 6,5,4,3,2,1 reducing, or 30µg as directed), and/or all courses with no dosing instructions, but unlikely to be maintenance therapy with a code for asthma or a lower respiratory event, where “maintenance therapy” is defined as: daily dosing instructions of <10µg prednisolone or prescriptions for 1mg prednisolone tablets.

^i^Lower respiratory consultations consist of the following: a) lower respiratory Read codes (including asthma, COPD and LRTI Read codes); b) asthma/COPD review codes excl. any monitoring letter codes; c) lung function and/or asthma monitoring; d) any additional respiratory examinations, referrals, chest x-rays or events.

^j^Fluticasone propionate (FP) equivalents.
