# Supplementary material for: Risk of pneumonia in obstructive lung disease: A real-life study comparing extra-fine and fine-particle inhaled corticosteroids
Source: PLoS One. 2017 Jun 15;12(6):e0178112. doi: 10.1371/journal.pone.0178112 (PMC5472262; doi:10.1371/journal.pone.0178112)
Supplement: S4 Table — (DOCX) [file pone.0178112.s005.docx]

S4 Table. Adjusted outcome results – pneumonia diagnosis by treatment group: fine vs. extra-fine particle in matched patients.

| Pneumonia diagnosis in outcome period | By treatment group | | Total | P-value^a^ |
| --- | --- | --- | --- | --- |
|  | **Fine-particle** | **Extra-fine particle** |  |  |
| Yes, n (%) | 31 (0.5) | 16 (0.2) | 47 (0.4) | 0.028 |
| No, n (%) | 6605 (99.5) | 6620 (99.8) | 13225 (99.6) |  |
| Total, n (%) | 6636 (100) | 6636 (100) | 13272 (100) |  |
| Unadjusted odds ratio (95% CI) | 1.00 | 0.50 (0.27, 0.93) |  | |
| Odds ratio (95% CI) adjusted for baseline confounders^b^ | 1.00 | 0.50 (0.25, 1.01) |  |  |

^a^Conditional logistic regression.

^b^Adjusted for COPD diagnosis (ever) (Y/N).
