# Supplementary material for: Risk of pneumonia in obstructive lung disease: A real-life study comparing extra-fine and fine-particle inhaled corticosteroids
Source: PLoS One. 2017 Jun 15;12(6):e0178112. doi: 10.1371/journal.pone.0178112 (PMC5472262; doi:10.1371/journal.pone.0178112)
Supplement: S5 Table — (DOCX) [file pone.0178112.s006.docx]

S5 Table. Adjusted outcome results – ATS/ERS exacerbations by treatment group: fine vs. extra-fine particle in matched patients.

| ATS/ERS exacerbations in outcome period | By treatment group | | Total | P-value^a^ |
| --- | --- | --- | --- | --- |
|  | **Fine-particle** | **Extra-fine particle** |  |  |
| 0, n (%) | 5112 (77.0) | 5319 (80.2) | 10431 (78.6) | <0.001 |
| 1, n (%) | 966 (14.6) | 861 (13.0) | 1827 (13.8) |  |
| 2+, n (%) | 558 (8.4) | 456 (6.9) | 1014 (7.6) |  |
| Total, n (%) | 6636 (100) | 6636 (100) | 13272 (100) |  |
| Rate ratio adjusted for baseline confounders^b^ | 1.00 | 0.90 (0.84, 0.97) |  | |

ATS: American Thoracic Society; ERS: European Respiratory Society.

^a^Conditional logistic regression.

^b^Adjusted for rhinitis diagnosis and/or therapy (Y/N), COPD diagnosis (ever) (Y/N) and number of prescriptions for SABA (categorised).
