# Supplementary material for: Risk of pneumonia in obstructive lung disease: A real-life study comparing extra-fine and fine-particle inhaled corticosteroids
Source: PLoS One. 2017 Jun 15;12(6):e0178112. doi: 10.1371/journal.pone.0178112 (PMC5472262; doi:10.1371/journal.pone.0178112)
Supplement: S6 Table — (DOCX) [file pone.0178112.s007.docx]

S6 Table. Adjusted outcome results – acute respiratory events by treatment group: fine vs. extra-fine particle in matched patients.

| Acute respiratory events in outcome period | By treatment group | | Total | P-value^a^ |
| --- | --- | --- | --- | --- |
|  | **Fine-particle** | **Extra-fine particle** |  |  |
| 0, n (%) | 4094 (61.7) | 4366 (65.8) | 8460 (63.7) | <0.001 |
| 1, n (%) | 1475 (22.2) | 1360 (20.5) | 2835 (21.4) |  |
| 2+, n (%) | 1067 (16.1) | 910 (13.7) | 1977 (14.9) |  |
| Total, n (%) | 6636 (100) | 6636 (100) | 13272 (100) |  |
| Rate ratio adjusted for baseline confounders^b^ | 1.00 | 0.90 (0.86, 0.95) |  | |

^a^Conditional logistic regression.

^b^Adjusted for rhinitis diagnosis and/or therapy (Y/N), COPD diagnosis (ever) (Y/N) and number of prescriptions for SABA (categorised).
