# Supplementary material for: Risk of pneumonia in obstructive lung disease: A real-life study comparing extra-fine and fine-particle inhaled corticosteroids
Source: PLoS One. 2017 Jun 15;12(6):e0178112. doi: 10.1371/journal.pone.0178112 (PMC5472262; doi:10.1371/journal.pone.0178112)
Supplement: S1 File — (DOCX) [file pone.0178112.s008.docx]

Online Supplement

## Definitions

### Severe Asthma Exacerbation - definition based on the ATS/ERS Task force definition

An exacerbation is defined as an occurrence of the following:

1. Asthma-related (Asthma**-**related includes all events with a **lower respiratory code**, i.e. all lower respiratory codes, including all asthma codes, and lower respiratory tract infection codes).
2. Hospital admissions OR
3. A&E attendance; OR
4. An acute course of oral corticosteroids

### Acute Respiratory Event

An event is defined as an occurrence of the following:

1. Asthma-related:
2. Hospital admissions OR
3. A&E attendance; OR
4. Acute use of oral corticosteroids; OR
5. Antibiotics prescribed with lower respiratory consultation (consisting of the following: a) Lower Respiratory Read codes (including Asthma, COPD and LRTI Read codes); b) Asthma/COPD review codes excl. any monitoring letter codes; c) Lung function and/or asthma monitoring; d) Any additional respiratory examinations, referrals, chest x-rays or events).

## Matched analyses

### Cohort definition

23,013 patients from the unmatched cohort were matched on the following criteria:

- Sex (M/F)
- Age (± 5 years)
- Baseline coding for pneumonia (Y/N)
- Smoking status (non-smoker / current smoker / ex-smoker)
- Number of acute courses of oral corticosteroids (0/1/2+)
- Number of prescriptions for antibiotics (0/1/2+)
- Average daily ICS dose at baseline (1-200/201-400/401-800/801+mcg; FP equivalent dose)
- Year of ICS step-up (± 3 years).

Randomisation led to 6,636 uniquely matched patient pairs (N=13,272). At baseline, matched patients in the extra fine-particle ICS cohort had lower ICS dose and average daily dose than matched patients in the fine-particle cohort (S3 Table).
